# Crosstalk between Shh and TGF-β Signaling in Cyclosporine-Enhanced Cell Proliferation in Human Gingival Fibroblasts

**DOI:** 10.1371/journal.pone.0070128

**Published:** 2013-07-26

**Authors:** Yi Chung, Earl Fu

**Affiliations:** 1 Graduate Institute of Life Sciences, National Defense Medical Center, Taipei, Taiwan, Republic of China; 2 Department of Periodontology, School of Dentistry, National Defense Medical Center and Tri-Service General Hospital, Taipei, Taiwan, Republic of China; Indiana University School of Medicine, United States of America

## Abstract

**Background:**

Immunosuppressant cyclosporine-A induces gingival hyperplasia, which is characterized by increased fibroblast proliferation and overproduction of extracellular matrix components and regulated by transforming growth factor-beta (TGF-β). The TGF-β and Sonic hedgehog (Shh) pathways both mediate cell proliferation. Crosstalk between these pathways in cancer has recently been proposed, but the hierarchical pattern of this crosstalk remains unclear. In normal fibroblasts, a TGF-β-stimulating Shh pattern was observed in induced fibrosis. However, Shh pathway involvement in cyclosporine-enhanced gingival proliferation and the existence of crosstalk with the TGF-β pathway remain unclear.

**Methodology/Principal Findings:**

Cyclosporine enhanced mRNA and protein levels of TGF-β and Shh in human gingival fibroblasts (RT-PCR and western blotting). A TGF-β pathway inhibitor mitigated cyclosporine-enhanced cell proliferation and an Shh pathway inhibitor attenuated cyclosporine-enhanced proliferation in fibroblasts (MTS assay and/or RT-PCR of PCNA). Exogenous TGF-β increased Shh expression; however, exogenous Shh did not alter TGF-β expression. The TGF-β pathway inhibitor mitigated cyclosporine-upregulated Shh expression, but the Shh pathway inhibitor did not alter cyclosporine-upregulated TGF-β expression.

**Conclusions/Significance:**

The TGF-β and Shh pathways mediate cyclosporine-enhanced gingival fibroblast proliferation. Exogenous TGF-β increased Shh expression, and inhibition of TGF-β signaling abrogated the cyclosporine-induced upregulation of Shh expression; however, TGF-β expression appeared unchanged by enhanced or inhibited Shh signaling. This is the first study demonstrating the role of Shh in cyclosporine-enhanced gingival cell proliferation; moreover, it defines a hierarchical crosstalk pattern in which TGF-β regulates Shh in gingival fibroblasts. Understanding the regulation of cyclosporine-related Shh and TGF-β signaling and crosstalk in gingival overgrowth will clarify the mechanism of cyclosporine-induced gingival enlargement and help develop targeted therapeutics for blocking these pathways, which can be applied in pre-clinical and clinical settings.

## Introduction

Cyclosporine A (CsA), a powerful immunosuppressant, is widely used to prevent organ rejection but has significant side effects in oral tissues; one of these side effects is gingival overgrowth, which is characterized by increased proliferation of fibroblasts, epithelial thickening, and overproduction of extracellular matrix components [1]–[4]. Various direct and/or indirect interactions between CsA and gingival fibroblasts have been investigated, including those that involve metabolic and synthetic activities [2], [5]–[8]. However, the molecular regulation of CsA-stimulated gingival overgrowth is not fully understood.

Transforming growth factor-beta (TGF-β) is a cytokine that regulates multiple cellular responses including cell proliferation, differentiation, senescence, and apoptosis [9], [10]. TGF-β seems to play a significant role in modulating the proliferation and/or migration of structural cells in the periodontium and in the production of different extracellular matrices by these cells [11]. Expression and secretion of TGF-β are upregulated in CsA-induced overgrown gingiva in humans and animals [1], [12]–[14]. CsA stimulates TGF-β production and restricts DNA synthesis via a TGF-dependent mechanism [15], [16]. However, TGF-β1 is unlikely to be the sole factor responsible for CsA-induced gingival overgrowth, because the difference in TGF-β1 levels in gingival cervical fluid between responding and non-responding overgrown sites are not statistically significant [17]. Thus, complex interactions between various mediators of tissue modeling may be involved in the pathogenic mechanisms of gingival overgrowth.

We previously demonstrated increased expression of cyclin D1 (hedgehog target gene), CDK4, and PCNA proteins in human gingival fibroblasts (HGFs) after CsA treatment [18]. Rb1 phosphorylation in HGFs was enhanced after treatment with CsA, which induced gingival cells to enter the G1/S phase transition and proceed to the DNA-synthesis phase, leading to cell proliferation [19]. Sonic hedgehog (Shh) is a member of the mammalian Hedgehog (Hh) family that plays a key role in embryogenesis, organogenesis, and adult tissue homeostasis [20]–[22]. Shh canonical signaling acts through the Patched (Ptc) and Smoothened (Smo) membrane proteins and induces transcriptional activation of the *Gli* gene. In the absence of Shh, Ptc maintains Smo in an inactivated state. After Shh binding, Ptc inhibition of Smo is released, and the signal is transmitted to promote transcription of Shh target genes, such as *Ptc* and *Gli* [23]–[25]. Shh signaling also controls, directly or indirectly, many target genes involved in cell proliferation, cell-fate determination, and tissue homeostasis [26]. However, the role of Shh in CsA-enhanced cell proliferation and overgrowth has not been fully elucidated.

Crosstalk between the TGF-β and Shh pathways in cancer has recently been proposed [27]. While the canonical signal transduction cascades of these pathways have been well characterized, there is increasing evidence that these pathways possess overlapping activities that challenge the efficiency of therapeutic targeting [28]–[33]. However, the crosstalk between the TGF-β and Shh pathways in CsA-enhanced cell proliferation has never been explored. In this study, we hypothesized that a crosstalk exists between Shh and TGF-β signaling in cyclosporine-enhanced cell proliferation, which is the major cause of cyclosporine-induced gingival hyperplasia. To test this hypothesis, we examined the impact of supplementation and inhibition of TGF-β or Shh on expression of Shh and TGF-β and CsA-enhanced cell proliferation in HGFs. Our aim was to test the hypothesis that the crosstalk exists between Shh and TGF-β signaling in CsA-enhanced cell proliferation; indeed, our results demonstrate this crosstalk exists and define a hierarchical pattern of crosstalk in which TGF-β regulates Shh expression in gingival fibroblasts.

## Materials and Methods

### Ethics Statement

HGF cell lines were obtained from the Coriell Institute for Medical Research. The Coriell Cell Repository maintains the consent and privacy of the donor of the fibroblast samples. Maintenance of all cell lines and all study protocols were in accordance with the guidelines approved by institutional review boards at the National Defense Medical Center.

### Cell Culture

HGFs were purchased from the Coriell Cell Repository (Camden, NJ, USA) (AG09319 and AG09429). Cells were cultured in Eagle’s MEM (InvitroGen, Grand Island, NY, USA) supplemented with 10% fetal bovine serum, 2 mM l-glutamine, and 100 U/mL antibiotics in plastic culture flasks and maintained at 37°C with 5% CO_2_. Once confluent, HGFs were trypsinized and replated on 10-cm or 6-well tissue culture dishes (Nunc AS, Roskilde, Denmark). Confluent fibroblasts were serum-starved overnight prior to each experiment. The HGFs used for the experiments were within 5 passages of one another. Cyclopamine, a steroidal alkaloid that specifically antagonizes the Shh signaling pathway through direct interaction with Smo [34] was purchased from Enzo Life Sciences, Inc. (Exeter, UK). TGF-β RI Kinase Inhibitor V, a TGF-β signaling inhibitor, was obtained from Calbiochem Inc. (San Diego, CA, USA).

### Antibodies and Reagents

Human recombinant Shh and TGF-β were purchased from R&D Systems (Minneapolis, MN, USA). Shh antibody was purchased from Santa Cruz Biotechnology (Santa Cruz, CA, USA) and anti-TGF-β and anti-α-tubulin antibodies were obtained from Abcam Inc. (Abcam, Cambridge, MA). The MCF-7 cell line was used as the positive control for Shh detection [35]. Relative densities were determined as the ratio of sample signal intensity to α-tubulin intensity.

### RNA Extraction and Reverse Transcription-Polymerase Chain Reaction (RT-PCR)

Total RNA was extracted from homogenized gingival tissue and fibroblasts. After reverse transcription of total RNA, PCR was performed as follows: initial denaturation at 94°C for 2 min 30 s, followed by 30 or 40 cycles at 94°C for 30 s, an appropriate annealing temperature (58–60°C) for 30 s, and 72°C for 55 s. PCR primers used were as follows: human Shh, sense (5′-ACCATTCTCATCAACCGGGT-3′) and antisense (5′-ATTTGGTAGAGCAGCTGCGA-3′), with an expected product of 269 bp; human TGF-β, sense (5′-GCGGTACCTGAACCCGTGTT-3′) and antisense (5′-GTCAATGTACAGCTGCCGCAC-3′), with an expected product of 477 bp; human Gli1, sense (5′-CAGAGAATGGAGCATCCTCC-3′) and antisense (5′-TTCTGGCTCTTCCTGTAGCC-3′), with an expected product of 285 bp; human PCNA, sense (5′-GCCGAGATCTCAGCCATATT-3′) and antisense (5′-ATGTACTTAGAGGTACAAAT-3′), with an expected product of 454 bp; and human glyceraldehyde-3-phosphate dehydrogenase (GAPDH), sense (5′-AGGTCGGAGTCAACGGATTTG-3′) and antisense (5′- GTGATGGCATGGACTGTGGT-3′), with an expected product of 532 bp. Amplified RT-PCR products were electrophoresed on a 1.5% agarose gel, visualized using SYBR® Safe DNA gel stain, and imaged on a Molecular Imager ChemiDoc™ XRS+ System with Image Lab™ Software (Bio-Rad Laboratories GmbH, Vienna, Austria). The relative densities were determined as the ratio of sample signal intensity to the intensity of the GAPDH band.

### Western Blot Analysis

After treatment with CsA, human recombinant Shh, or TGF-β1, the gingival fibroblasts were harvested, lysed with NP-40 lysis buffer, and the lysate was boiled. Protein concentrations were determined using the BCA™ Protein Assay Reagent Kit (Pierce, Rockford, IL, USA). Total cell lysates were separated by sodium dodecyl sulfate-polyacrylamide gel electrophoresis and transferred onto a polyvinylidene difluoride membrane. After blocking with 5% skim milk for 1 h, the membranes were hybridized with one of the following: polyclonal anti-Shh antibody (Santa Cruz Biotechnology, Santa Cruz, CA, USA), monoclonal anti-TGF-β1 antibody (Abcam, Cambridge, UK), or monoclonal anti-α-tubulin antibody (Epitomics, Burlingame, CA, USA). After washing with PBST, the blots were incubated with a horseradish peroxidase-conjugated secondary antibody and the bands were visualized by enhanced chemiluminescence [36] using the Molecular Imager ChemiDoc™ XRS+ System with Image Lab™ Software (Bio-Rad Laboratories GmbH, Vienna, Austria). HT-29, SW480, and MCF-7 cell lines served as positive controls for Shh and TGF-β1 [35]. Relative densities were determined as the ratio of sample signal intensity to α-tubulin intensity.

### MTS Cell Proliferation Assay

HGFs were placed in 96-well plates containing Eagle’s MEM supplemented with 10% fetal bovine serum and cultured to 60–70% confluence. The cells were washed once with phosphate-buffered saline (PBS), and the medium was replaced with Eagle’s MEM for serum starvation overnight. Before and after treatment with CsA (0, 300, 500, 800, or 1000 ng/mL in 50% dimethylsulfoxide), recombinant Shh (0, 50, 100, 300, 500, or 1500 ng/mL in sterile PBS containing 0.1% bovine serum albumin), or recombinant TGF-β (0, 0.1, 0.5, 1, 2.5, 3, 5, or 10 ng/mL in 4 mM HCl containing 1 mg/mL bovine serum albumin) for 24 and 48 h, cell proliferation was tested using the MTS (3-[4,5-dimethylthiazol-2-yl]-5-[3-carboxymethoxyphenyl]-2-[4-sulfophenyl]-2H-tetrazolium, inner salt) assay (CellTiter 96®AQueous One Solution; Promega, Madison, WI, USA). The effect of CsA, Shh, or TGF-β on gingival fibroblast proliferation was compared by assessing fibroblast proliferation before and after treatment according to a modified version of a previous method [19]. The dosage of TGF-β pathway inhibitor was adopted from previous studies [37], [38].

### Statistical Analysis

Student’s *t*-test was used to evaluate relative differences between the control and CsA groups in their Shh, TGF-β, PCNA, and Gli mRNA levels and Shh and TGF-β protein levels (relative intensity). One-way analysis of variance and Duncan’s *post hoc* tests were used to evaluate the effect of CsA, recombinant Shh, and recombinant TGF-β on cell proliferation in HGF cultures in comparison to control, CsA-treated, and CsA plus inhibitor (cyclopamine or TGF-β RI Kinase Inhibitor V)-treated groups, as well as the expression of Shh, TGF-β, PCNA, and Gli mRNAs and Shh and TGF-β protein levels. A *p*-value of <0.05 was considered statistically significant.

## Results

### Role of TGF-β in Cell Proliferation of HGFs upon CsA Administration

**Figure 1:**
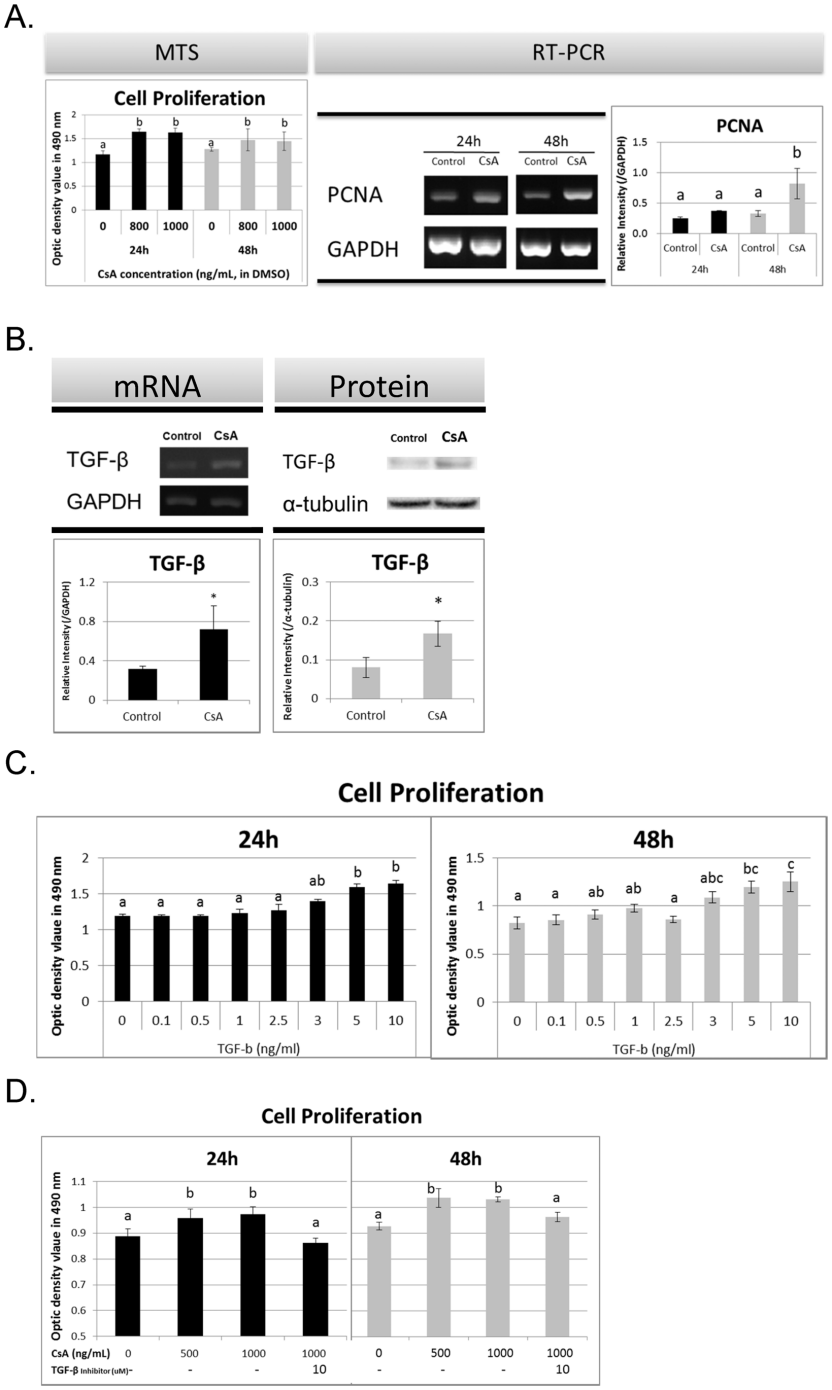
Role of TGF-β in HGF Proliferation after CsA Treatment. (A) CsA enhanced cell proliferation by MTS assay and RT-PCR analysis of PCNA. (B) CsA increased TGF-β mRNA and protein expressions, examined by RT-PCR (24 h) and Western blotting (48 h). (C) TGF-β enhanced cell proliferation and (D) inhibition of TGF-β mitigated the CsA-enhanced cell proliferation. Experiments were repeated 3 times. Data are expressed as mean and SD; *significantly different from the control group at *p*<0.05 by Student’s *t*-test or one-way ANOVA; and a–b, subsets obtained after *post hoc* analysis.).

The effect of CsA on HGF cell proliferation was assessed using the MTS assay. The absorbance values significantly increased in HGFs treated with CsA relative to that in HGFs treated with DMSO, regardless of the concentration (800 and 1000 ng/mL) or time point (24 and 48 h) (Figure 1A). Moreover, PCNA mRNA expression was significantly upregulated after 48-h treatment with 1000 ng/mL CsA.

TGF-β mRNA and protein expression significantly increased in HGFs after treatment with CsA (1000 ng/mL) (Figure 1B). Exogenous TGF-β significantly enhanced cell proliferation in a dose-dependent manner (Figure 1C). However, TGF-β inhibition attenuated CsA-enhanced cell proliferation (Figure 1D).

### Effect of Shh on HGF Proliferation upon CsA Administration

**Figure 2:**
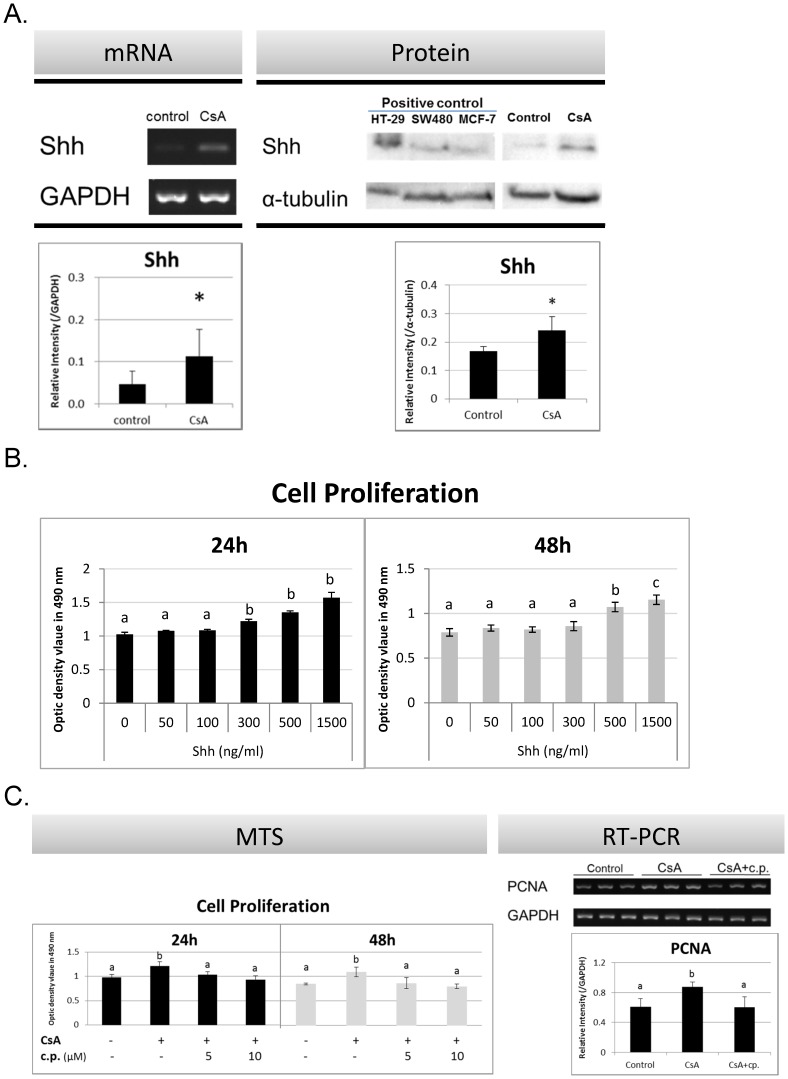
Role of Shh in HGF Proliferation after CsA Treatment. (A) Shh mRNA and protein levels were upregulated in HGFs after CsA treatment (1000 ng/mL) at 48 h and 72 h, respectively. (B) Shh enhanced cell proliferation and (C) inhibition of Shh attenuated CsA-enhanced cell proliferation (C). HT-29, SW480, and MCF-7 cell lines served as positive controls. Experiments were repeated 3 times. Data are expressed as mean and SD; *significantly different from the control group at *p*<0.05 by Student’s *t*-test or one-way ANOVA; a–c, subsets obtained after *post hoc* analysis.

Shh mRNA and protein expression significantly increased in HGFs after CsA (1000 ng/mL) treatment (Figure 2A). Exogenous Shh enhanced cell proliferation in a dose-dependent manner (Figure 2B), while inhibition of Shh mitigated the CsA-enhanced MTS absorbance and PCNA mRNA expression (Figure 2C).

### TGF and Shh Signaling Molecule Expression after TGF or Shh Supplementation in HGFs

**Figure 3:**
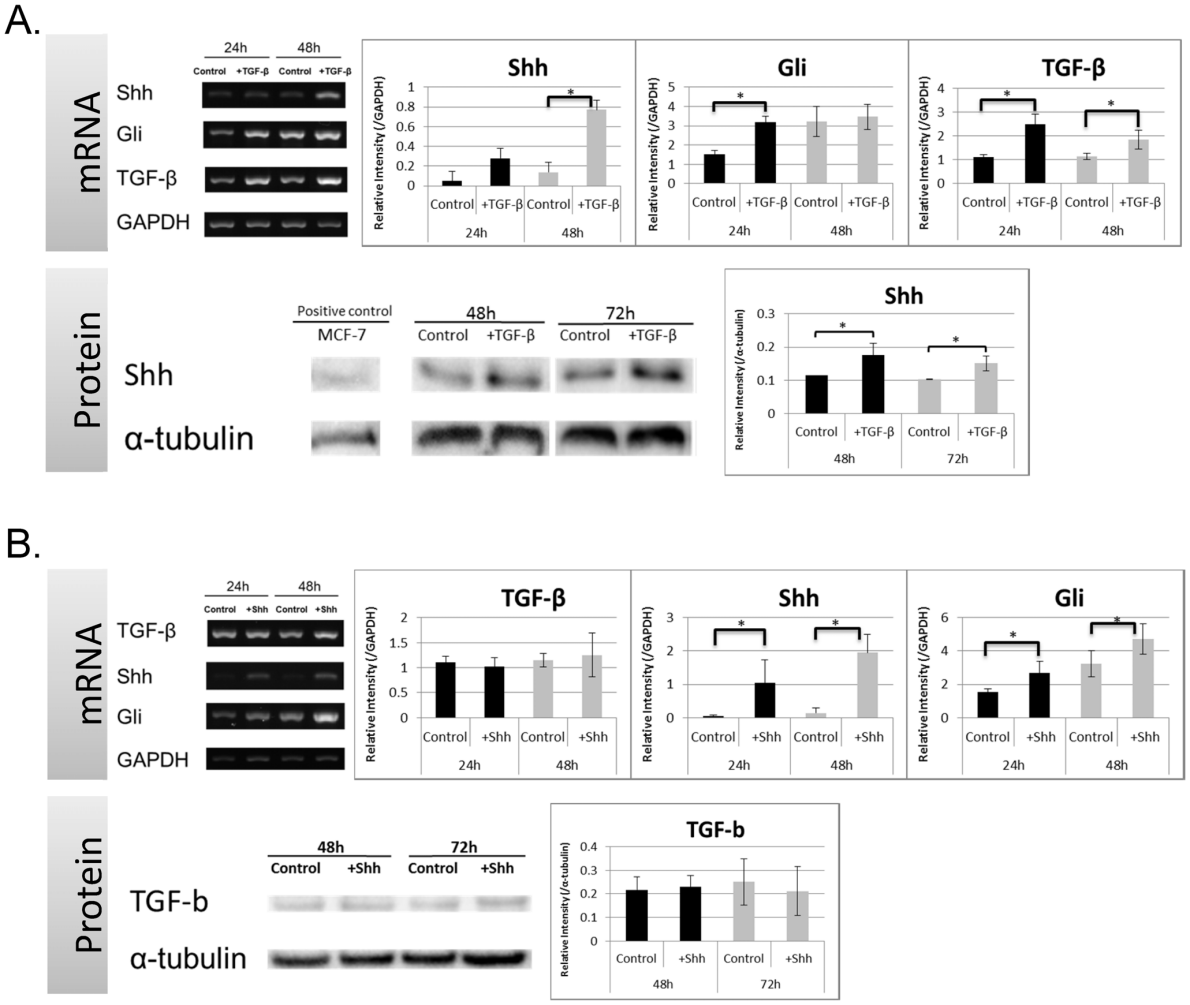
Expression of TGF-β and Shh Signaling Molecules with Exogenous Supplementation in HGFs. (A) Exogenous TGF-β upregulated mRNA expression of Shh, Gli, and TGF-β by RT-PCR and protein expression of Shh by Western blotting. (B) Exogenous Shh upregulated Shh and Gli mRNA and protein expression, but did not alter TGF-β mRNA expression. Experiments were repeated 3 times. Data are expressed as mean and SD; *significantly different from the control group at *p*<0.05 by Student’s *t*-test).

CsA enhanced cell proliferation and enhanced transcription of Shh and TGF-β simultaneously; we sought to determine whether a crosstalk between Shh and TGF-β exists in CsA-enhanced gingival proliferation. To determine whether TGF-β signaling is involved in CsA-enhanced Shh expression, we treated HGFs with exogenous TGF-β and characterized Shh expression. Shh (48 h), Gli (24 h), and TGF-β (24 and 48 h) mRNA significantly increased in HGFs treated with exogenous TGF-β (Figure 3A). Significantly elevated Shh protein expression in HGFs (24 and 48 h) in the presence of exogenous TGF-β was confirmed by western blotting.

However, treatment with exogenous Shh did not alter TGF-β mRNA expression at 24 and 48 h in comparison to untreated cells, although Shh and Gli mRNA levels were significantly enhanced (Figure 3B). In addition, TGF-β protein levels did not change after Shh supplementation (24 and 48 h).

### TGF and Shh Signaling Molecule Expression after TGF or Shh Inhibition

**Figure 4:**
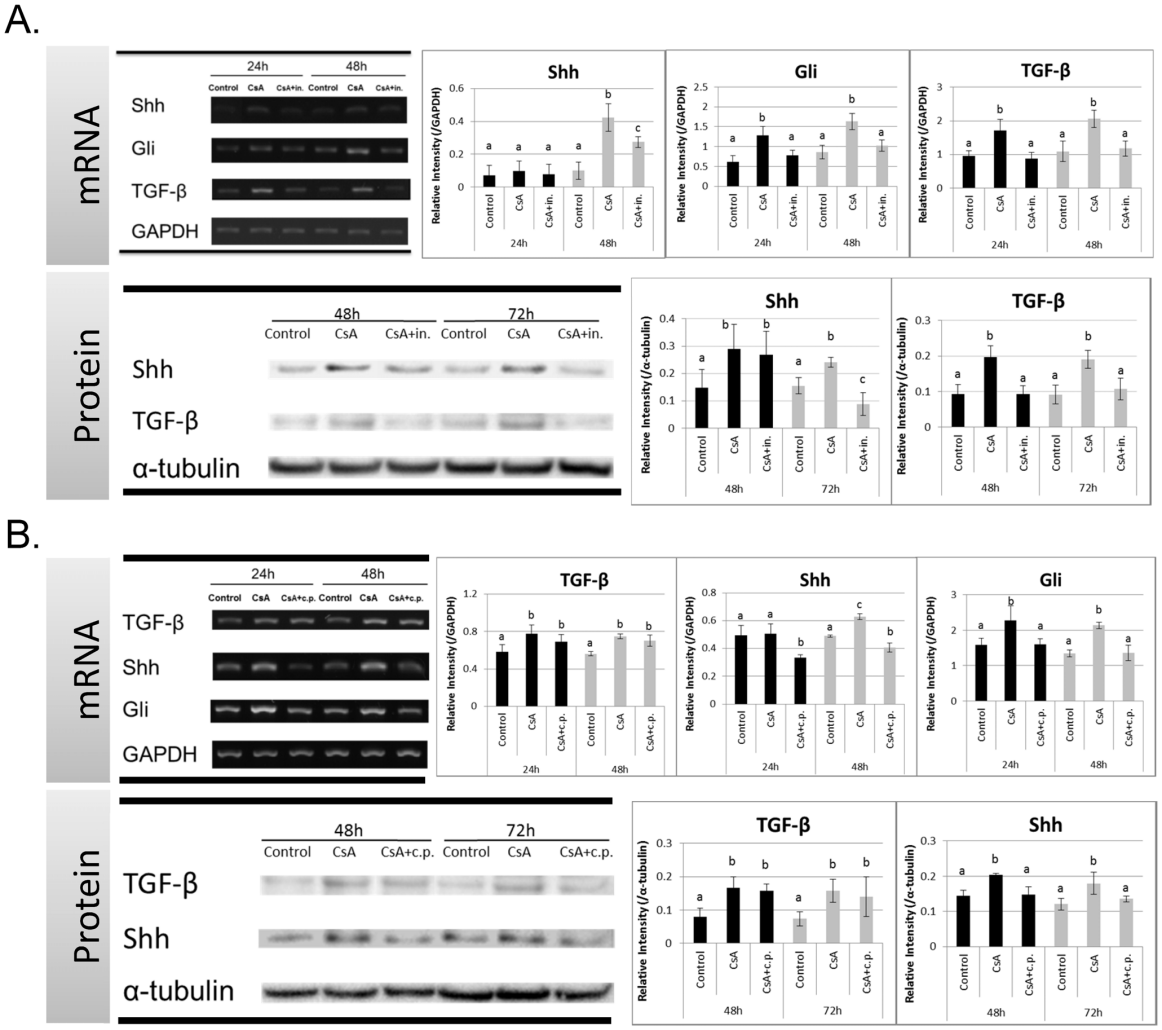
Expression of TGF and Shh Signaling Molecules after TGF-β or Shh Inhibition. (A) TGF-β signaling inhibitor, ALK5 kinase inhibitor, mitigated CsA-upregulated mRNA expression of Shh, Gli, and TGF-β by RT-PCR and protein expression Shh and TGF-β by Western blotting. (B) Shh signaling inhibitor, cyclopamine, did not alter CsA-upregulated TGF-β expression, but it did attenuate CsA-upregulated Shh expression. Experiments were repeated 3 times. Data are expressed as mean and SD by one-way ANOVA; a–c, subsets obtained after *post hoc* analysis.

To verify whether TGF-β signaling is involved in CsA-enhanced Shh expression, we treated HGFs with a TGF-β-specific inhibitor and assessed Shh expression. Shh mRNA expression (48 h) in cells that were treated with CsA and/or the TGF-β signaling inhibitor differed significantly (Figure 4A). CsA significantly increased Shh mRNA expression; however, this effect was reduced by TGF-β signaling inhibitor. Similar findings were observed for Gli and TGF-β expression at 24 and 48 h (Figure 4A). Significantly lower Shh and TGF-β protein levels were observed at 48 and 72 h in cells treated with CsA and the TGF-β signaling inhibitor compared to that in cells treated with CsA alone (Figure 4A).

To verify whether Shh signaling is involved in CsA-enhanced TGF-β expression, HGFs were treated with an Shh-specific inhibitor. TGF-β mRNA expression in the 3 treatment groups (control, CsA, and/or Shh signaling inhibitor) differed significantly (Figure 4B). Although CsA significantly enhanced TGF-β mRNA expression, there was no difference between cells treated with CsA or CsA plus Shh inhibitor. CsA-enhanced Shh and Gli mRNA expression was significantly reduced after treatment with Shh signaling inhibitors, although TGF-β protein levels were unaffected.

## Discussion

The prevalence of gingival enlargement induced by the immunosuppressant CsA has been reported by many transplant centers, and it varies from 7% to 80% depending on reporting criteria. A thorough review of well-controlled studies suggests the overall prevalence is 25%–30% [39]. The TGF-β and Shh pathways are known to regulate cell proliferation; however, Shh expression in CsA-enhanced gingival cell proliferation has never been explored. Our results demonstrate that CsA upregulates *Shh* and *TGF-β* gene expression in HGFs (Figures 1 and 2). Although varied patterns of crosstalk between these pathways in cancer cells have been proposed [27], [40], our results indicate a hierarchical pattern of crosstalk. This pattern suggests TGF-β regulates Shh expression in CsA-enhanced gingival cell proliferation, because treatment with exogenous TGF-β increased Shh expression, and inhibition of TGF-β signaling mitigated cyclosporine-upregulated Shh expression; however, TGF-β expression was unchanged regardless of the addition or inhibition of Shh (Figures 3 and 4). This hierarchical pattern of crosstalk between Shh and TGF-β signaling is consistent with evidence that TGF-β upregulates Shh in normal fibroblasts and tumor cells [30], [31], [41].

**Figure 5:**
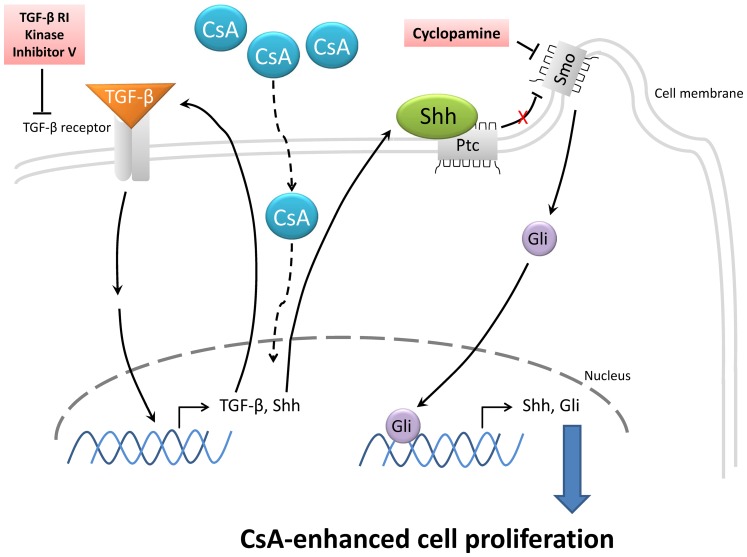
Model of Crosstalk between Shh and TGF-β Signaling in CsA-Enhanced Cell Proliferation. CsA-enhanced cell proliferation in HGFs *via* Shh signaling is modulated by TGF-β. The schematic diagram is generated, according to our findings from this study. CsA may upregulate the Shh expression directly or indirectly *via* TGF-β signaling. Increased Shh expression leads to Gli activation and contributes to HGF proliferation. TGF-β RI kinase inhibitor V and cyclopamine inhibit the TGF-β and Shh pathways, respectively.

This study explored the role of TGF-β, and our findings were consistent with those of other studies (Figure 1C and 1D) [1], [13], [37], [38], [42]. However, the expression and role of Shh in CsA-enhanced gingival cell proliferation has never been examined. Inhibition of the Shh pathway reduced gingival fibroblast proliferation, whereas treatment with exogenous Shh increased cell numbers (Figure 2B and 2C), indicating that Shh plays an important role in enhancing gingival cell proliferation. TGF-β signaling inhibition significantly reduced CsA-upregulated Shh expression, but the Shh signaling inhibitor had no significant effect on CsA-upregulated TGF-β expression; therefore, we suggest that TGF-β upregulates Shh and leads to CsA-enhanced Shh expression and cell proliferation in fibroblasts (Figure 5).

Shh and TGF-β signals control various aspects of embryonic development and cancer progression. Although their canonical signal transduction cascades have been well characterized, there is increasing evidence that these pathways possess overlapping functions that challenge efficient therapeutic targeting [27]. A number of scenarios suggest crosstalk between the TGF-β and Shh pathways in cancer. For example, epithelial tumor cells chronically exposed to TGF-β1 exhibit Shh upregulation and signal induction and acquisition of the epithelial–mesenchymal transition phenotype, which is responsible for tumor cell aggressiveness and metastasis [31]. In addition, upregulation of TGF-β signaling in systemic sclerosis may drive activation of Shh signaling in fibrotic murine skin and cultured human skin fibroblasts [30]. Moreover, TGF-β promoted survival of immature liver cells and stimulated surviving mature hepatocytes to release Shh [41].

While TGF-β likely contributes to some of the biological effects of Shh, it is also likely that the opposite is true. For example, Shh promotes motility and invasiveness of gastric cancer cells through TGF-β-mediated activation of the ALK5-Smad3 pathway [32]. Other reports indicate that TGF-β regulates events downstream of Smo, independent of Shh. For example, the TGF-β signaling pathway appears to be critical for Smo-mediated basal cell carcinoma development [29]. In addition, TGF-β is a potent transcriptional regulator of Gli2, which may activate Gli1 independent of Shh signaling [28]. Moreover, it has been recently discovered that TGF-β inhibits PKA activity while inducing Gli2 and Gli1 expression [43]. PKA blockade may contribute to an increase in the pool of full-length activator Gli proteins, thus inducing an Shh response. These reports indicate that TGF-β and Shh signaling may form a vicious cycle that promotes and amplifies the metastatic process, whereas Gli2 and its downstream target Gli1 may play a major role in promoting tumor cell invasion and resistance to apoptosis.

Although numerous studies have investigated tumor cells, few were performed in normal cells, as we have shown here. Remarkably, a recent study using normal fibroblasts showed that TGF-β receptor type I drives activation of Shh signaling in cultured fibroblasts and murine skin, suggesting a hierarchical system in which TGF-β signaling stimulates hedgehog signaling in induced fibrosis [30]. These data are consistent with our results.

Interestingly, we also observed that treatment with exogenous TGF-β or Shh increased gene expression of the counterpart components, and inhibition of TGF-β or Shh signaling in CsA-treated HGFs attenuated counterpart gene expression. This finding suggests autocrine stimulatory and inhibitory signaling roles for TGF-β and Shh in CsA-enhanced gingival fibroblast proliferation. These data were supported by the findings of previous studies that showed the autocrine effect of Shh in cancer and myofibroblastic hepatic stellate cells [44], [45], as well as the autocrine effect of TGF-β in CsA-treated human gingival fibroblast proliferation [14], [42].

This is the first study to demonstrate the role of Shh and the crosstalk between Shh and TGF-β in CsA-enhanced gingival cell proliferation. We also define a hierarchical pattern of crosstalk in which TGF-β regulates Shh expression in gingival fibroblasts (non-cancerous). In conclusion, CsA increased proliferation in HGFs, and this proliferation was blocked by Shh and TGF-β inhibitors. Exogenous TGF-β enhanced expression of Shh signaling molecules; however, exogenous Shh or Shh blockade did not affect TGF-β expression. We, therefore, propose that CsA-enhanced gingival cell proliferation could be partially affected by Shh, which might be modulated by upregulation of TGF-β. The vast majority of publications that describe the roles of TGF-β or Shh signaling in cancers and cell proliferation have only assessed these cytokines in isolation. It is important for future studies to simultaneously evaluate the contribution of both pathways to identify proper targets for therapeutic intervention at a given stage of disease progression. CsA-enhanced gingival fibroblast proliferation could also be used as a model to investigate the relationship between the TGF-β and Shh pathways. With the discovery of crosstalk between Shh and TGF-β, we hope to shed light on the mechanism of CsA-induced gingival enlargement and provide clues for development of targeted therapeutics aimed at blocking either of these pathways, which can be used in pre-clinical and clinical settings.
